# Till death do us part: amyotrophic lateral sclerosis in the ICU

**DOI:** 10.1186/cc13223

**Published:** 2014-03-17

**Authors:** Y Valzani, A Marudi, G De Grandis, J Mandrioli, S Baroni, R Stacca, E Bertellini

**Affiliations:** 1 University of Modena and Reggio Emilia, Modena, Italy; 2 Nuovo Ospedale Civile Sant'Agostino Estense, Modena, Italy

## Introduction

The aim of the ICU is to give the best life care to the admitted patients in order to preserve and restore the patients' quality of life. In some critical patients, life-supporting therapies become actions to support the end stage of their life [1]. This is the paradox of the ICU. Patients affected by amyotrophic lateral sclerosis (ALS) belong to this category: for these patients, all medical actions do not improve the quality of life but just prolong it. ALS or Charcot's disease is the most common motor neuron disease which causes severe motor disability and evolves in a few years to death [2]. We want to present our experience in patients affected by ALS in the ICU.

## Methods

We collected data about patients affected by ALS admitted to the ICU from 1 January 2010 to 31 October 2013. We considered entry diagnosis, mean age, need to perform tracheostomy and/or percutaneous endoscopic gastrostomy (PEG), and mortality in the ICU.

## Results

Sixty patients were admitted for neuromuscular respiratory failure complicated by aspiration pneumonia. Mean age was 43 years. The male to female ratio was 3:1. All patients underwent percutaneous tracheostomy and PEG. One of them died in the ICU because of septic shock. In every case we honestly communicated the worsening of the disease and we perceived the awareness of it by the patients and/or their family. In no case did patients ask us to withdraw the necessary cures such as percutaneous tracheostomy and PEG. In no case did they ask us to die. In Europe the ‘end of life care' law is very dyshomogeneous because of different cultures, traditions, religions, and beliefs. In Italy euthanasia is not allowed by law. It is the physician often by himself that has to make the final decision according to the patient's mind.

## Conclusion

Our experience shows that patients affected by severe motor disability are not always willing to die. It is essential that decisions should be patient-centered and taken multidisciplinarily, based on open and emphatic communication, involving family and caregivers.
